# Fluid Pressure Monitoring-Based Strategy for Delicate Grasping of Fragile Objects by A Robotic Hand with Fluid Fingertips

**DOI:** 10.3390/s19040782

**Published:** 2019-02-14

**Authors:** Toshihiro Nishimura, Yosuke Suzuki, Tokuo Tsuji, Tetsuyou Watanabe

**Affiliations:** 1 Graduated school of Natural science and Technology, Kanazawa University, Kanazawa 9201192, Japan; 2 Faculty of Frontier Engineering, Institute of Science and Engineering, Kanazawa University, Kanazawa 9201192, Japan; suzuki@se.kanazawa-u.ac.jp (Y.S.); tokuo-tsuji@se.kanazawa-u.ac.jp (T.T.)

**Keywords:** soft robots, robotic hands, delicate grasping, fluid pressure monitoring

## Abstract

In this study, we propose a strategy for delicately grasping fragile objects using a robotic gripper with highly deformable fluid fingertips. In an earlier study, we developed a soft fingertip, referred to as a fluid fingertip, which was fabricated from a deformable rubber bag filled with incompressible fluid. The proposed strategy detects the preferable grasping point where fracturing of the target object is avoided while the applied force or pressure from the fluid fingertip is substantially transmitted to the target. In this grasping point, the behavior of the fluid pressure changes with respect to the pushing distance when pushing at a constant speed. The other features of the strategy determining the grasping point are as follows: (1) the threshold for the detection of the grasping point is fixed or constant with respect to the type of target object; (2) information regarding the deformation and stiffness of the fingertips and target object is not required. The detection of the grasping point through behavioral changes in the pressure is performed by comparing the fitting accuracies of fitting models utilizing information of the fluid pressure and pushing distance. The validity of the proposed approach is verified through several experiments.

## 1. Introduction

**Figure 1:**
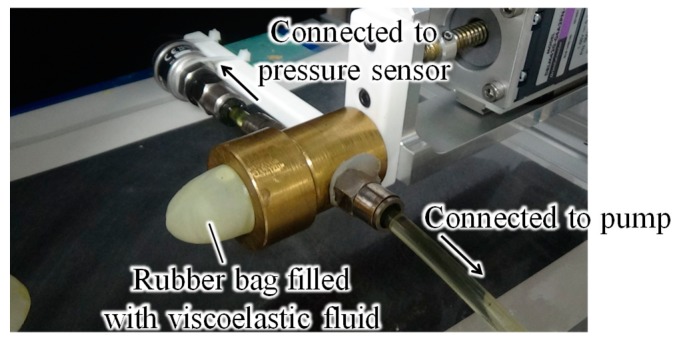
Fluid fingertip.

There are specific requirements for robotic systems working in human environments. Robotic hands play an important role as end-effectors in such robotic systems, and many types of robotic hand have been developed [1,2]. In a human environment, interactions between humans and robots cannot be avoided, and thus, the surfaces of robots must be soft to avoid physical damage during these interactions. Considering this, we previously developed a robotic hand with a soft surface by filling a rubber bag with a viscoelastic fluid (Figure 1) and demonstrated that the hand can grasp a wide variety of fragile objects [1,2,3]. We call this fingertip a fluid fingertip. Owing to the fluid incompressibility, a uniform contact pressure distribution is obtained. Unevenness in an object’s shape can be accommodated by the fluid deformation. These factors are beneficial for delicately grasping fragile objects. In particular, we presented a methodology for grasping (kinugoshi) tofu, which is a highly soft and fragile object, without using any advanced knowledge regarding the fracture stress. We focused on the pressure profile of the filled fluid because this corresponds to the stress. We showed that tofu could be grasped without fracturing at the point where the behavior of the fluid pressure changed [4]. However, the significance of this point was unclear. In addition, the most important aspect of how to grasp an object using such highly deformable fingertips was not considered. The present study proposes an extended version of our previous grasping strategy for a robotic hand with highly deformable fingertips [4]. In this study, the following features of the grasping strategy are revealed:

1. The grasping point corresponds to the point at which the stress inside the object becomes activated over the whole inner area, and deformation begins at the free boundaries. In addition, at this point, invasion of the object body owing to compression at the contact area is almost eliminated, and a substantial grasping pressure or force is transmitted to or exerted on the object.
2. Fractures and cracks can be avoided.
3. The threshold for detecting the grasping point is constant regardless of the object.
4. Information on the deformation and stiffness of the fingertips and target object is unnecessary for detecting the grasping point.

Our previous strategy [4] began from the state at which the fluid fingertip was in contact with the target object. In actual cases, grasping normally starts from a state where the fingertips and object are sufficiently separated. The new strategy deals with the case in which only fluid pressure monitoring is utilized.

The proposed strategy adopts the following assumptions: the speed of compressing or pushing the target object with the highly deformable fingertips is constant, and the fingertips have high surface friction. The remainder of this paper is organized as follows. The next subsection describes related research. Section 2 describes the compression test for examining the behavior of the highly deformable fingertips. Section 3 presents the methodology for detecting the grasping point online. Section 4 presents experimental results demonstrating the validity of the proposed approach.

### Related Research

Anthropoid robotic hands have been developed in consideration of their human affinity (see References [5,6,7,8,9,10,11]). Normally, the surface of a robotic finger is rigid during use in factories and is not suitable for adaptation to the human environment. Shimoga and Goldenberg claimed that gel is effective for constructing the surfaces of fingertips because it can reduce the contact impact and strain energy and conforms itself to the shape of an object [12]. In addition to these advantages, we observed further benefits of gel in our previous studies, including uniform contact pressure distribution and automatic increase in stiffness [1]. The main drawback of using gel to construct fingertips is the limitation on the maximum applicable force. Therefore, a two-layer structure in which a rigid component is installed inside the gel was presented in References [1,3,4,13].

Fitting to the target object’s shape is key for achieving universal grasping (where a wide variety of objects can be grasped by a robotic hand). The snake-like gripper presented by Hirose and Umetani can be considered a pioneering approach in this regard [14]. Furthermore, Kim and Song developed a gripper that included hybrid variable stiffness actuators, which could control the contact stiffness [15]. Brown et al. [16] developed a universal gripper for grasping objects with a wide variety of shapes, which was based on the jamming phenomenon [2]. Choi and Koc also proposed a design for inflatable rubber pockets on the gripping sides of robotic hands [17] to grasp objects with a wide variety of shapes. Pettersson et al. utilized a magnetorheological (MR) fluid to construct a gripper that adjusts to the object shape and provides space to confine objects [18]. This space was formed by molding the MR fluid. Kim et al. [19] presented a soft skin for safe interaction between children and robots. The research results showed that a robotic hand with soft skin could grasp several objects, including a plastic cup and a roll of paper. In the above studies, the robotic hands could grasp several fragile objects, including eggs and fruits.

However, the optimal time at which the compression applied on the target object (being grasped with a highly deformable (fluid) fingertip) must be stopped has not been considered. In other words, a grasping strategy without altering the deformable part to become rigid via the jamming transition has not been considered. The present study aims to investigate this issue.

## 2. Compression Test Using Fluid Fingertip

### 2.1. Structure of Fluid Fingertip

As described above, the fluid fingertip (Figure 1) was constructed using a rubber bag filled with a viscoelastic fluid. The radius of each fingertip was 11 mm. Nitrile was used as the outer rubber material and the fluid used was oil (chainsaw oil, ISO VG 100 (International Organization for Standard Viscosity Grade)). A screw clamp was employed to improve the sealing performance. A pressure sensor (KEYENCE, AP-12s) was connected using a tube. Additionally, a pump was connected using a tube to control the fluid pressure. According to Pascal’s law, if a fluid is incompressible, then the pressure is the same everywhere, even when an external pressure or force is applied to the container used to confine the fluid. In addition, a change in the fluid volume indicates a change in the fluid pressure. Therefore, by controlling the piston we could control the fluid pressure. The main body was constructed using brass to prevent corrosion. Owing to the deformability of the fingertip, its shape conformed to the object shape. As we demonstrated in our previous paper [1], the contact pressure distribution was uniform. These two features made the system highly effective for grasping fragile objects. The fingertip stiffness increased with an increase in the deformation. The main difference between the fluid fingertip and similarly designed fingertips of robotic hands, such as jamming grippers [16], is the inner media. Liquid is utilized in the fluid fingertip, whereas rigid particles are utilized in jamming grippers. Therefore, the deformability of the fluid fingertip is higher than that of jamming grippers. Large contact area, correspondingly large frictional force, high adaptability to the object’s shape, and high absorption ability of contact impacts can then be obtained with a small grasping force. Hence, the fluid fingertip is preferable to a jamming gripper for grasping fragile or complex-shaped objects. Conversely, in comparison with jamming grippers, the fluid fingertips cannot provide high stiffness, correspondingly cannot apply a large grasping force, and thus, cannot grasp heavy objects.

### 2.2. Experimental Setup

**Figure 2:**
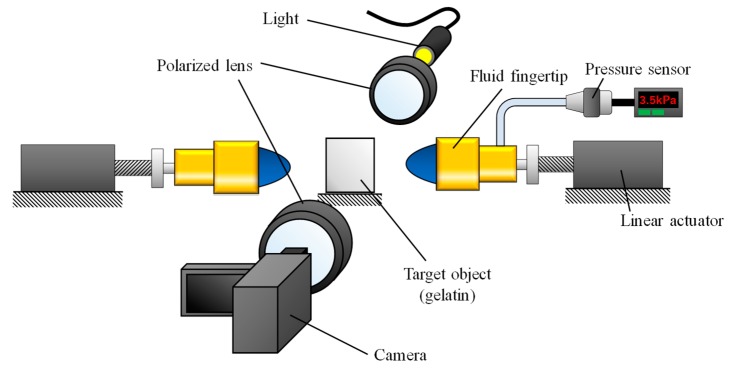
Schematic view of the experimental setup for the compression test with photoelastic imaging.

Compression tests were conducted to examine the behavior of the fluid pressure when pushing the fluid fingertip against a soft object. Figure 2 shows the experimental setup for the compression tests. To simulate the grasping event, two fingers were utilized for the test. The compression speed $v_{g}$ was set to 2 mm/s. The fluid pressure p was measured by the pressure sensor. The object and the fingertips were initially set up in such manner that the distance between each fingertip and the object was 10 mm. To examine the stress distribution during the compression, photoelastic imaging [20] was performed. For the photoelastic imaging, gelatin, which is a photoelastic material, was utilized as the target object. A plane polariscope setup was utilized for the photoelastic imaging, as illustrated in Figure 2. The arrangement for the setup consisted of a white light source (MITSUTOYO Megalight100), first linear polarizer (SIGMAKOKI SPF-50C-32), investigated target, second linear polarizer (SIGMAKOKI SPF-50C-32), called the analyzer, and video camera (SONY FDR-AX55) for recording. The directions of the two polarizers were orthogonal to each other. In this setup, a transparent or photoelastic material is optically isotropic under the absence of stress, whereas it undergoes birefringence when stress is applied. A fringe pattern corresponding to the induced birefringence can be observed. When utilizing a white light source, both isoclinic and isochromatic fringes are observed. The isochromatic fringe pattern is colorful, and corresponds to the difference in principal stresses, or equivalently, the maximum shear stress. The isoclinic fringe pattern is dark and corresponds to the directions of the principal stresses. This study focuses on the isochromatic fringe pattern, showing the stress distribution. The number of fringes in the isochromatic pattern is associated with the magnitudes of the principal stresses. A high number of color bands in the isochromatic fringe pattern indicates a high stress or stress concentration. The main purpose of photoelastic imaging is to understand the general phenomenon. Therefore, we focus not on the quantified values of the induced stress, but on the transitions of the stress distribution and concentration (namely, the color pattern).

### 2.3. Results and Discussion

**Figure 3:**
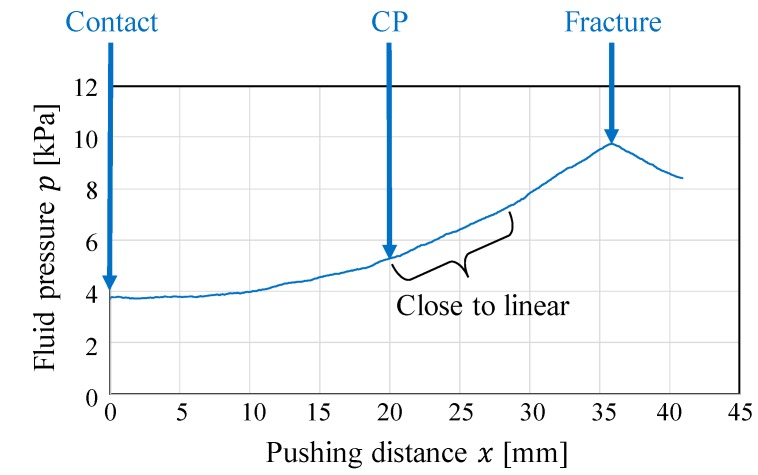
Behavior of the fluid pressure in the compression test.

**Figure 4:**
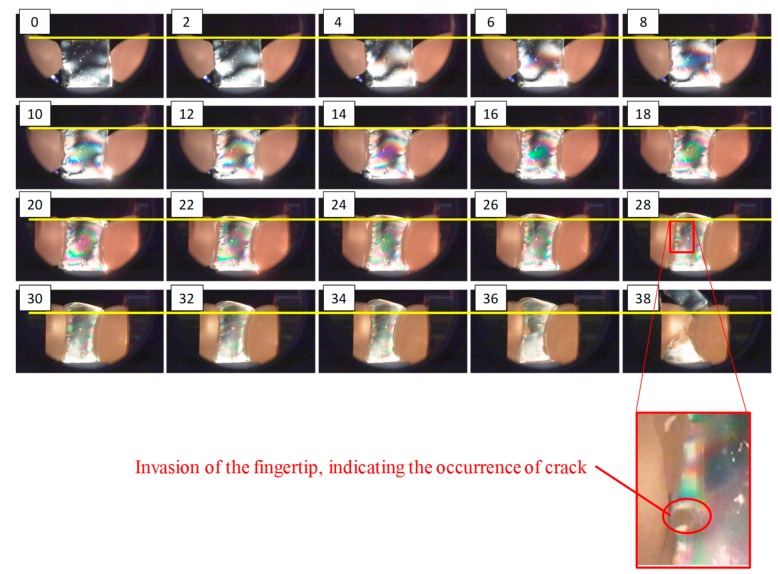
Snapshots of the photoelastic images during the compression test. The number at the upper left of each image is the corresponding pushing distance x [mm] from the occurrence of contact between the object and fingertip. The yellow line indicates the position of the top surface of the object in the initial state.

The pressure behavior results are illustrated in Figure 3, where x [mm] denotes the pushing distance from the point where the contact between the object and fingertips occurred. At first, the pressure increased nonlinearly. As the pushing distance increased, the fluid pressure behavior changed from nonlinear behavior to almost linear behavior. In this study, this point is referred to as the “changing point” (CP). Figure 4 presents snapshots of the photoelastic images during the compression test. The number at the upper left of each image indicates the corresponding pushing distance x (mm). The yellow line indicates the position of the top surface of the object at the initial state.

At first, the fluid fingertips invaded into the object (gelatin) and the rate of increase in pressure was extremely low. As the pushing distance x (mm) increased, the internal stress spread out, as shown from the photoelastic images. Around the CP (x=20 mm), the morphology or shape change in the stress distribution (the change in the positions and thicknesses of the color bands) became small, and deformation started around the free boundaries. The degree of invasion of the fluid fingertips became almost constant. The corresponding pressure behavior became close to linear (see Figure 3).

When the change in pressure is small, the change in pressure in the thermodynamics of fluid mechanics is defined as

(1) $$dp=K\frac{dV}{V}$$

Here, p denotes the pressure, V denotes the volume, and K denotes the bulk modulus of elasticity. If the rate of volume change $\frac{dV}{V}$ is constant, then the following relation can be obtained:

(2) dp=const

Equation (2) indicates that if the shape deformation proceeds with a constant volume change, then the change in pressure can be linear. The linear pressure behavior around the CP corresponds to the state represented by equation (2). As shown in Figure 4, the degree of invasion of the fingertips became close to constant around the CP, and thus, the rate of decrease of the fluid volume became almost constant and the state became close to that represented by Equation (2). The change in volume of the object body without considering the invasion of the fingertips was small before x=18 mm, whereas it became clear after the CP. Correspondingly, the color patterns around the free boundaries in the photoelastic images became constant at this point, and the deformation or extension at the free boundaries became clear. This corresponds to the invasion of the fluid fingertips almost stopping. Additionally, it indicates that the applied pressure from the fluid fingertips was transmitted to the free boundaries, i.e., the applied pressure was substantially transmitted to the object body.

Following the linear behavior, the rate of increase in pressure behavior changed. Around x=28 mm (around the area where the pressure behavior ceased being linear), part of the left fingertip invaded the object body (see Figure 4). This invasion is a sign of a crack or fracture. Therefore, the grasping should be conducted before the linear behavior stops.

The analyses at the CP are summarized as follows:

- The invasion of the object body owing to compression at the contact areas almost stops, and the applied pressure or force for grasping is substantially transmitted to or exerted on the object.
- Fractures and cracks can be avoided.

Therefore, the CP is the preferable grasping point, and the preferred grasping methodology is to detect the CP, stop the compression, and pick up the object to be grasped. The subsequent section will present the methodology for detecting the CP.

## 3. Methodology for Detecting the Grasping Point

Here, we describe the methodology for detecting the CP online. Because photoelastic imaging can only be applied to photoelastic or transparent materials, using only a pressure sensor to measure the inner fluid pressure is preferable. This is also more cost-effective than using force sensors. Pressure sensors can be connected to the tube, which is connected to the fluid part, to measure the inner fluid pressure, whereas force sensors require installation space and transmission devices. In addition, the pressure information is highly similar to the force information because the fluid fingertip provides a uniform contact pressure, as shown in [1]. We present a strategy considering the following requirements:

(1) The CP can be detected online.
(2) CP detection can be conducted by monitoring the fluid pressure only.
(3) The threshold for the detection is constant with respect to the type of target object.
(4) Advanced knowledge about the stiffness and deformation of the target object is not required.

**Figure 5:**
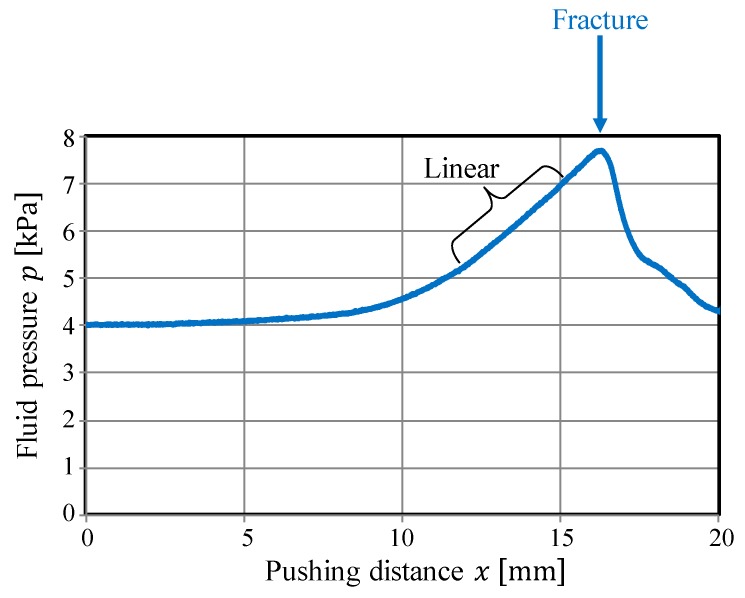
Behavior of the fluid pressure in the compression tests for soft tofu [4].

To detect the CP, we adopted a methodology wherein the point at which the behavior change is detected by comparing the fitting accuracies of different simple and complex regression models, in a manner similar to that in References [4,13]. A methodology in which a linear section, indicating a sign of a crack or fracture, is detected by monitoring the fitting accuracy of the linear approximation could be adopted. As shown in Figure 5, the linear section when compressing tofu is clear, and a methodology based on the linear approximation could be valid. Therefore, we adopted the approach in Reference [4]. However, Equation (2) represents the ideal case, and the actual pressure behavior is not always completely linear, as suggested by Figure 3. In such a case, threshold tuning is required, which indicates that the material properties of the target object must be known in advance. In addition, the behavioral change at the CP is not always clear. Hence, we adopted a methodology based on comparing the fitting accuracies of simple and complex models. Before the CP, the data arrangement will be simple, and both the simple and complex models can provide good fitting accuracies. In contrast, if the data arrangement deviates from the original one at the CP, then the fitting accuracy in the simple model will decrease while that in the complex model can be maintained. Hence, the difference in the fitting accuracies of the simple and complex models significantly increases at the CP. By detecting this increase, the CP can be detected. The main benefit of the methodology based on comparing the fitting accuracies of different models is that the threshold for detecting the CP does not have to be changed according to the type of object, and thus, can be constant. The original data arrangement includes a curve, and two- and three-dimensional polynomial functions are then prepared as approximations for the simple and complex models, respectively.

**Figure 6:**
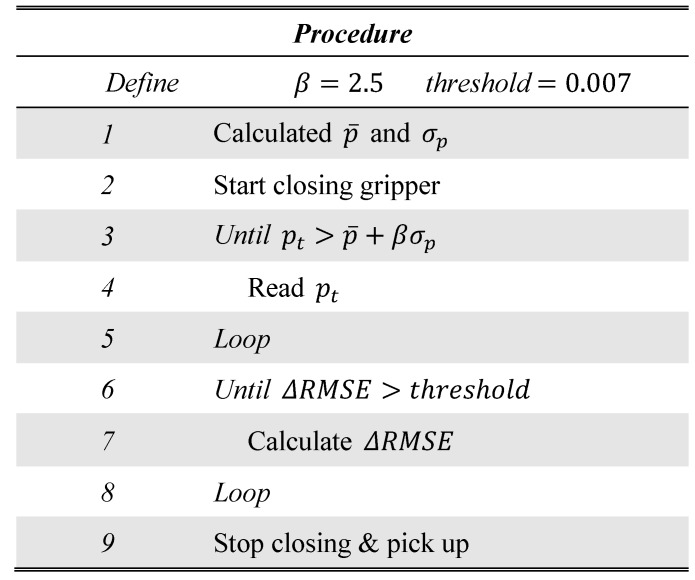
Procedure of the proposed strategy for detecting the CP: $\bar{p}$ and $\sigma_{p}$ denote the average and standard deviation, respectively, of the fluid pressure when the fingertip is unloaded and β is a tuning parameter. *Threshold* is the threshold for ΔRMSE, which is the difference between the root mean square errors (RMSEs) when fitting with the two- and three-dimensional polynomial functions.

We refer to the point at which the fluid pressure begins to increase as the starting point (SP). The steps of the strategy can be summarized as follows: First, we detect the SP, and subsequently, we detect the CP. The procedure of this strategy is illustrated in Figure 6.

Let $p_{t}$ denote the fluid pressure at time t. Consequently, the SP can be regarded as the point at which $p_{t}$ begins to increase. We detected the SP by verifying whether the following condition is satisfied:

(3) $$p_{t}>\bar{p}+\beta \sigma_{p}$$

Here, $\bar{p}$ and $\sigma_{p}$ denote the average and standard deviation, respectively, of the fluid pressure when the fingertip is unloaded and β is a tuning parameter. The threshold ($\bar{p}+\beta \sigma_{p}$) is determined by the system settings and is independent of the target object and pushing speed.

Subsequently, to detect the CP we adopted the methodology mentioned above. The two- and three-dimensional polynomial functions were assumed to represent the simple and complex fitting models, respectively. The regression started from the SP and was performed each time new data were obtained. Let $t_{i}$ denote the time for the $i^{\mathrm{th}}$ time step. The two- and three-dimensional polynomial functions at the $i^{\mathrm{th}}$ time step are defined as follows:

(4) $$\begin{array}{cc} {\hat{p}}_{t}^{i2}= & \sum_{j=0}^{2}\alpha_{j}^{i2}t^{j}\\ {\hat{p}}_{t}^{i3}= & \overset{3}{\underset{j=0}{\sum }}\alpha_{j}^{i3}t^{j} \end{array}$$

Here, $\alpha^{i2}={\left[a_{0}^{i2},a_{1}^{i2},a_{2}^{i2}\right]}^{T}\;\mathrm{and}\;\alpha^{i3}={\left[a_{0}^{i3},a_{1}^{i3},a_{2}^{i3},a_{3}^{i3}\right]}^{T}$ are the coefficients of the respective functions. At the $N^{\mathrm{th}}$ time step, we have a dataset $D_{t_{N}}=\left\{\left(t_{1},p_{t_{1}}\right),\left(t_{2},p_{t_{2}}\right),\ldots ,\left(t_{N},p_{t_{N}}\right)\right\}$, where $p_{t_{i}}$ denotes the obtained fluid pressure value corresponding to the (uniform) contact pressure at the *i*^th^ time step. In this case, the coefficients $\alpha^{i2}$ and $\alpha^{i3}$ are obtained by solving the following simultaneous linear equations (see the Appendix A) with little computational effort:

(5) $$\begin{array}{cc} \alpha^{i2}= & \underset{\alpha^{i2}}{\mathrm{argmin}}\sum_{j=1}^{N}{\left|p_{t_{j}}-{\hat{p}}_{t_{j}}^{i2}\right|}^{2}\\ \alpha^{i3}= & \underset{\alpha^{i3}}{\mathrm{argmin}}\overset{N}{\underset{j=1}{\sum }}{\left|p_{t_{j}}-{\hat{p}}_{t_{j}}^{i3}\right|}^{2} \end{array}$$

To compare the fitting accuracies of the models, the root mean square error (RMSE) was calculated. Let $RMSE_{i2}$ and $RMSE_{i3}$ denote the RMSEs for the two- and three-dimensional polynomial functions, respectively, at the *i*^th^ time step. These are defined as follows:

(6) $$\begin{array}{cc} {\mathrm{RMSE}}_{i2}= & \overset{N}{\underset{j}{\sum }}\sqrt{\frac{{\left(p_{t}{}_{j}-{\hat{p}}_{t_{j}}^{i2}\right)}^{2}}{N}}\\ {\mathrm{RMSE}}_{i3}= & \overset{N}{\underset{j}{\sum }}\sqrt{\frac{{\left(p_{t}{}_{j}-{\hat{p}}_{t_{j}}^{i3}\right)}^{2}}{N}} \end{array}$$

Subsequently, the difference in the fitting accuracies at the $i^{\mathrm{th}}$ time step, $\Delta RMSE_{i}$, is defined as follows:

(7) $$\Delta RMSE_{i}=RMSE_{i2}-RMSE_{i3}$$

**Figure 7:**
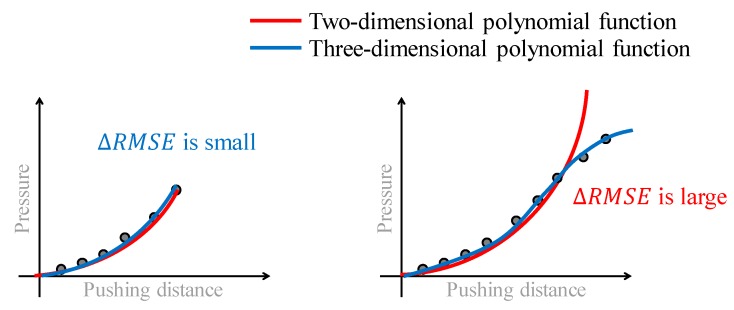
CP detection method.

As shown in Figure 7, the two- and three-dimensional polynomial functions were selected as the simple and complex models, respectively, in such manner that their fitting accuracies before the CP are close to each other and $\Delta RMSE_{i}$ is small. Around the CP, the data are rearranged and the curve expressing the behavior from the SP becomes complex. Therefore, the simple model cannot easily express the curve whereas the complex model can. In this case, ΔRMSE becomes large. Hence, by checking whether the value of ΔRMSE exceeds a threshold value (0.007 in this case), the CP can be detected. The two prepared models do not have to describe the data very well, because the purpose is only to detect the change in the data arrangement. The CP detection method requires a simple model that cannot describe the change in the data arrangement very accurately, and a complex model that can achieve this better than the simple model. Therefore, the simple two- and three-dimensional polynomial functions perform well.

## 4. Experimental Validation

**Figure 8:**
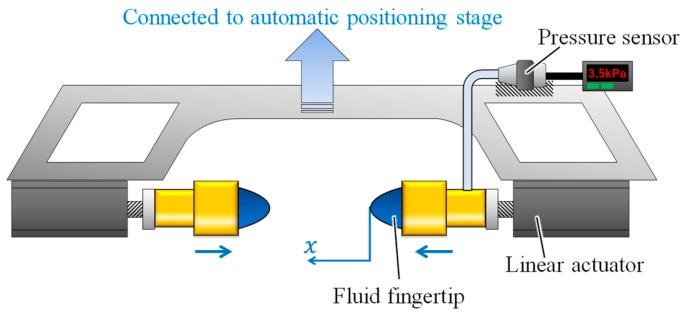
Experimental setup for grasping test.

To verify the effectiveness of the proposed strategy, grasping tests were conducted on various objects. Figure 8 illustrates the experimental setup for the grasping tests. A robotic gripper was utilized, consisting of two opposing linear actuators (oriental motor and DRLM42G-04A2PN-K) with one fluid fingertip attached to each of them. According to the proposed grasping strategy, a pressure sensor (Keyence, AP-12S) was installed on one of the fluid fingertips to monitor the fluid pressure. The gripper was mounted on an automatic vertical positioning stage.

**Figure 9:**
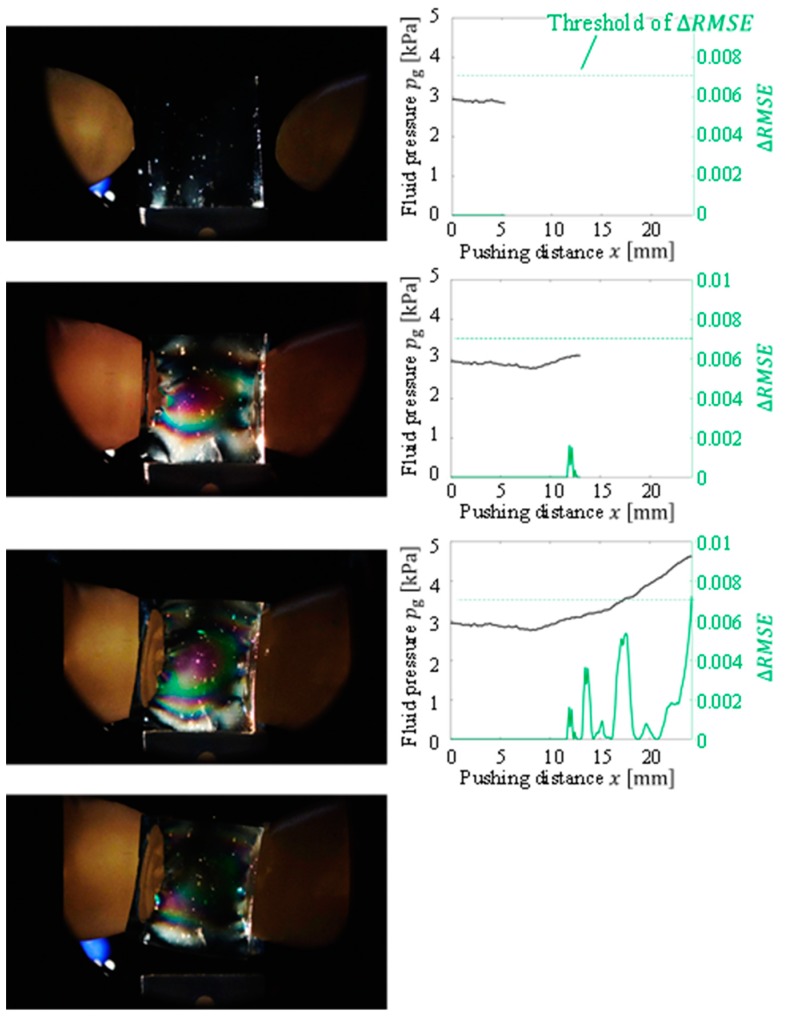
Overview of the grasping test (gelatin target).

**Figure 10:**
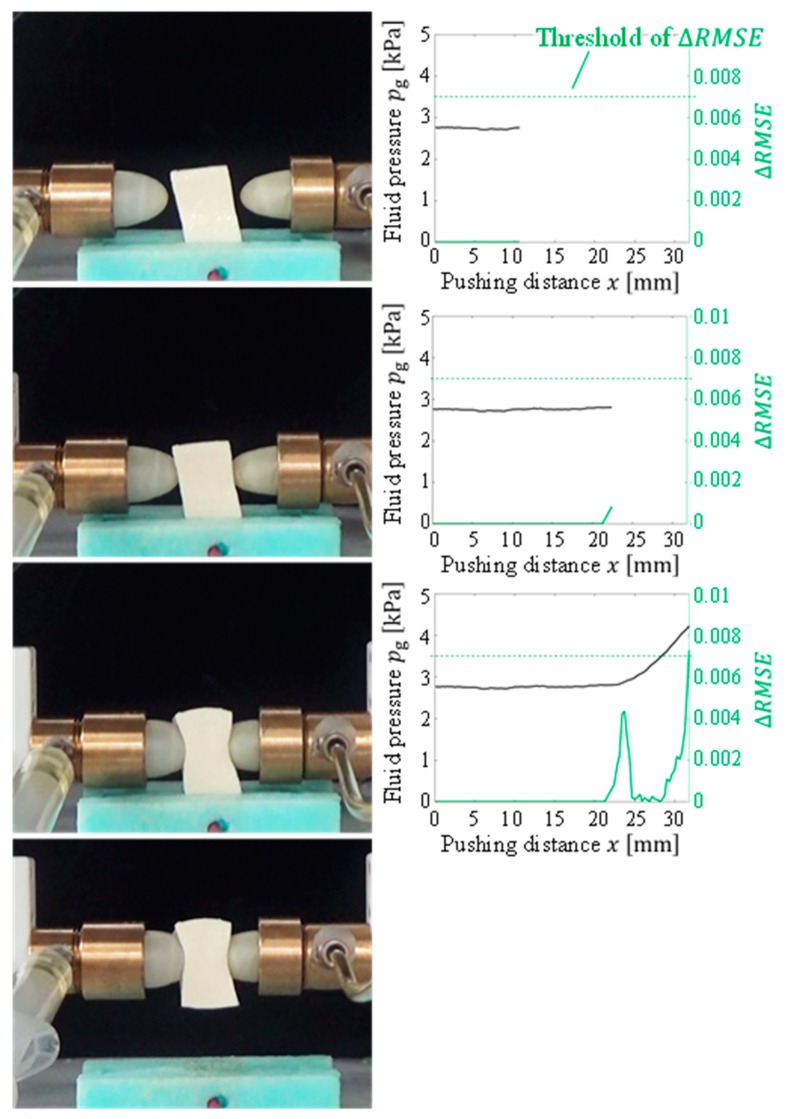
Overview of the grasping test (soft tofu target).

**Figure 11:**
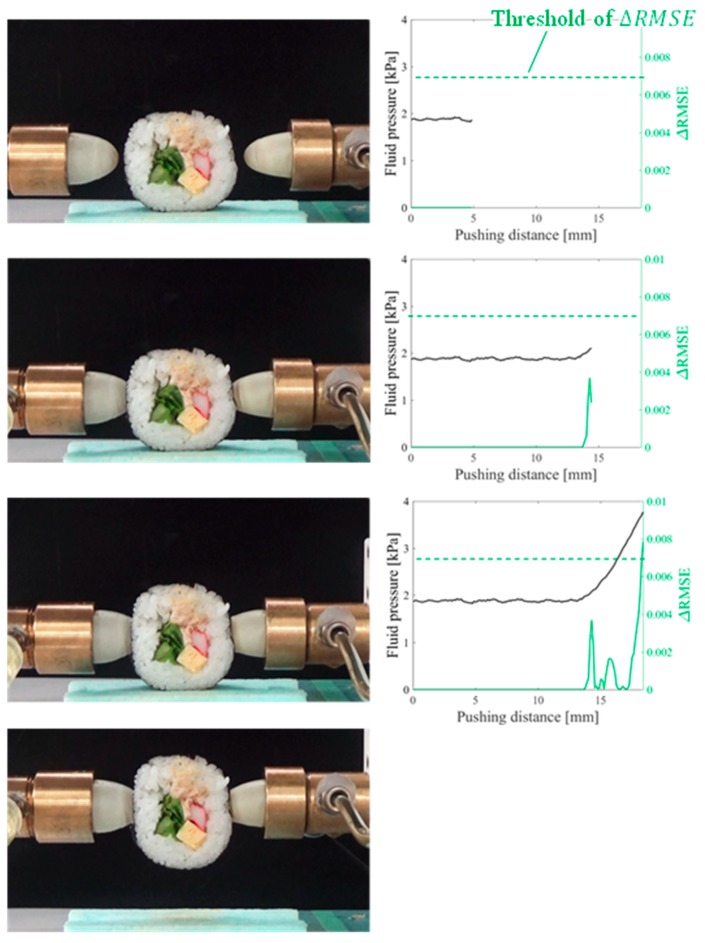
Overview of the grasping test (sushi target).

**Figure 12:**
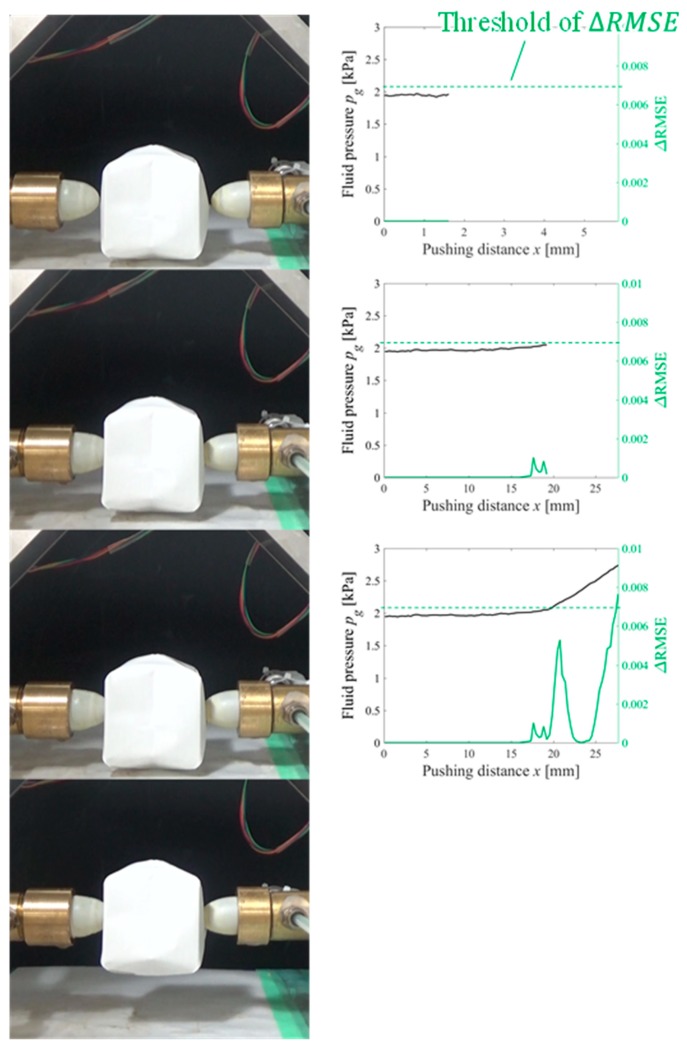
Overview of the grasping test (origami target).

**Figure 13:**
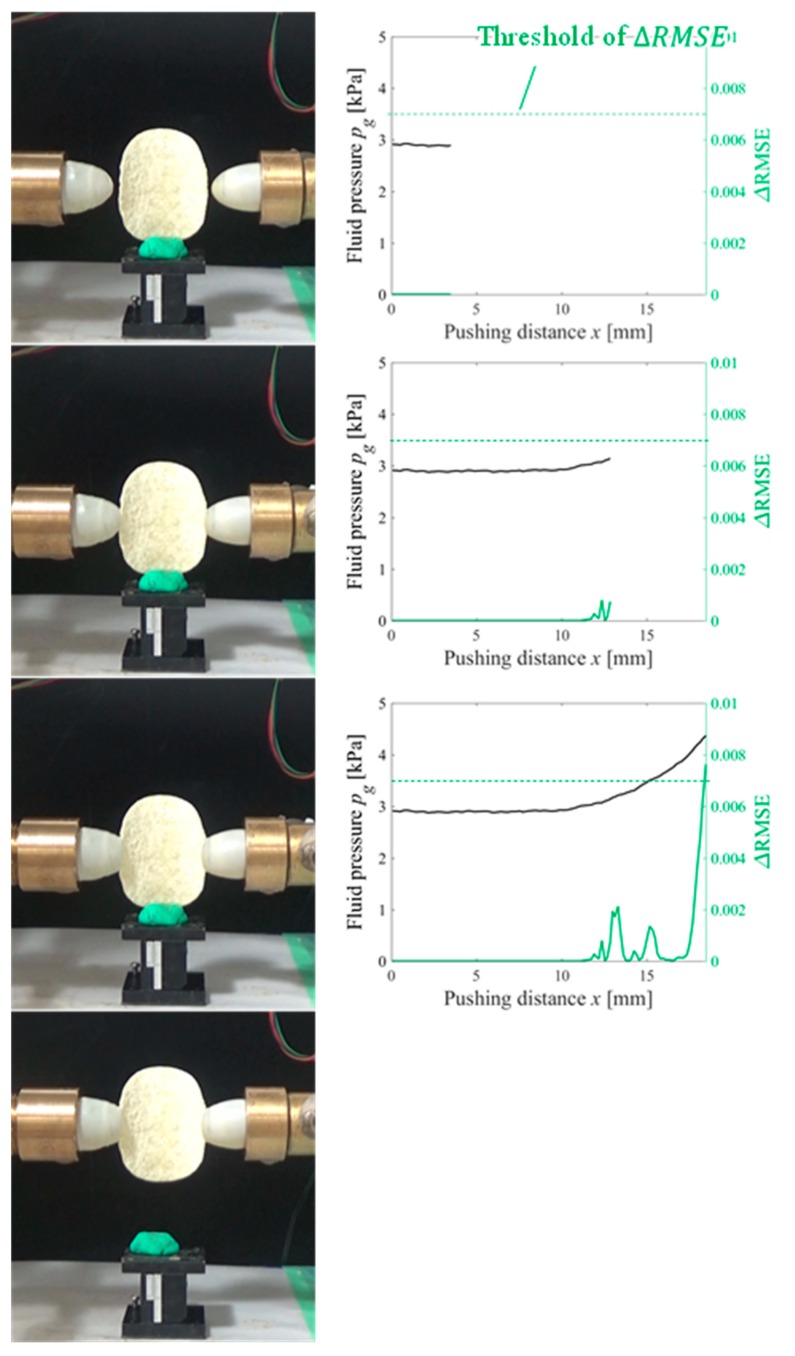
Overview of the grasping test (potato chip target).

Gelatin, soft tofu, sushi, origami [21], and potato chips were selected as the target objects. The target objects were placed at the center, between the fingertips. The origin of the pushing distance x was randomly set such that the object was not contacted by both fingertips at the origin. The tests were conducted five times for each target object. All the objects were grasped without sustaining any damage. Figure 9, Figure 10, Figure 11, Figure 12 and Figure 13 present images of the grasping tests. When grasping the gelatin, photoelastic imaging was adopted to confirm the absence of cracks and activation of shape deformation around the free boundaries at the detected CP. Although every target was fragile, no fractures were observed in the target objects. Small shape deformations around the free boundaries were observed when each of the objects was grasped. These deformations indicate a substantial transmission of the applied pressure or force during grasping. Hence, the proposed strategy allows fragile objects to be grasped without using any prior knowledge of the fracture stress.

**Figure 14:**
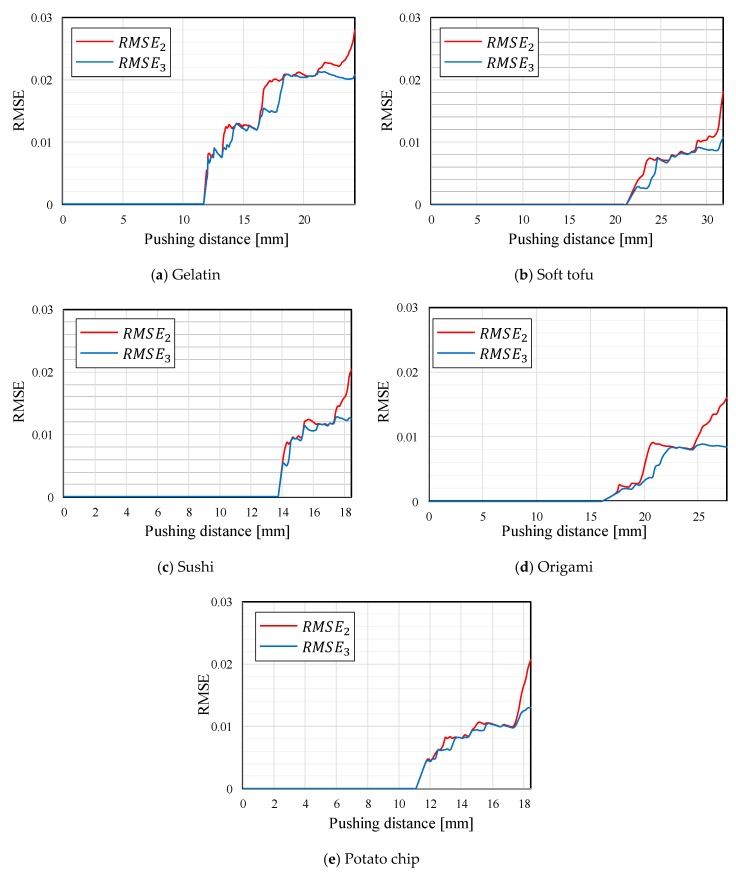
Behaviors of $RMSE_{2}$ and $RMSE_{3}$ in the grasping test for each target object.

Figure 9, Figure 10, Figure 11, Figure 12 and Figure 13 indicate that the pressure value at the CP varies with the type of target object, and it is difficult to detect the CP using the same threshold for the pressure value. Figure 14 illustrates the behaviors of $RMSE_{2}$ and $RMSE_{3}$ between the SP and CP in the grasping test for each target object. The value of $RMSE_{2}$ at the CP varied with the type of object (for example, 0.027 for gelatin and 0.015 for origami). If we attempt to detect the CP by only checking the value of $RMSE_{2}$, the threshold should be defined according to the type of target. This means that we require prior knowledge of the target object. In contrast, the proposed strategy was capable of detecting the appropriate grasping point for each target with a unique threshold for the difference in RMSE. The utilization of the unique threshold indicates that prior knowledge about the type of target is unnecessary. The results validate the effectiveness of the proposed strategy.

**Figure 15:**
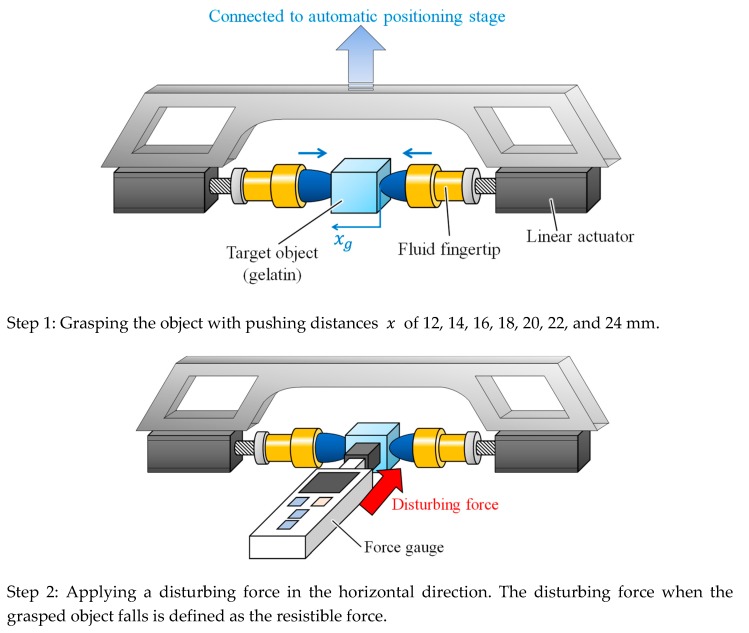
Experimental setup for investigating the resistible force when grasping with various degrees of compression.

**Figure 16:**
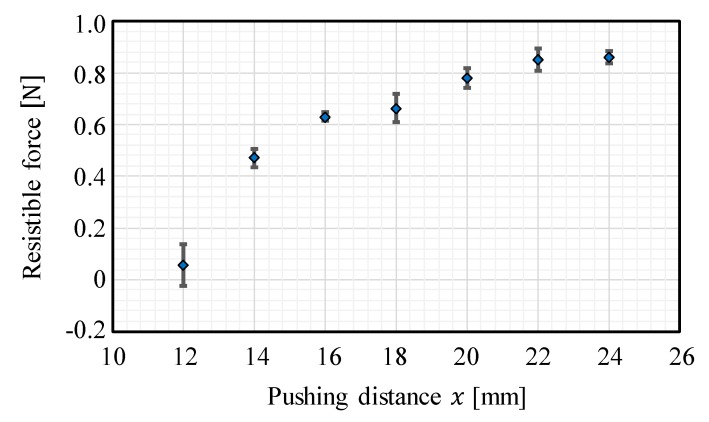
The resistible force when grasping with various degrees of compression: the average values and standard deviations are shown for each pushing distance.

To verify the significance of the CP from the viewpoint of grasping stability, we investigated the resistible force when grasping the gelatin object with the fluid fingertips under various degrees of compression. The experimental setup is illustrated in Figure 15, where a force gauge (IMADA DST-50N) was added to the setup shown in Figure 8. The object was first grasped with the two opposing fluid fingertips with pushing distances of 12, 14, 16, 18, 20, 22, and 24 mm (Step 1 in Figure 15). The values of the pushing distance correspond to those in Figure 4, and the pushing distance of 20 mm corresponds to the CP. Subsequently, a load in the horizontal direction was applied to the side of the object and we increased the value of this load until the object fell. The maximum value of the applicable load was defined as the resistible force and examined. The experiment was conducted three times for each case. Figure 16 illustrates the results, where the average value and standard deviation are displayed for each pushing distance. Around the CP (the pushing distance of 20 mm), the resistible force converged to a high value. Moreover, the standard deviation of the resistible force converged to a small value around the CP. The grasping was maintained even when a disturbing force was applied, indicating stable grasping. Therefore, a high resistible force indicates highly stable grasping and the stability of the grasping maximized around the CP. Additionally, the applied force was substantially transmitted to the object at the CP. The reason for the convergence of the resistible force is that the invasion into the object body almost stopped and the contact area became almost constant. When the pushing distance x was less than the CP, the resistible force was small, and it varied or fluctuated. This indicates that grasping at this pushing distance is more unstable than that at the CP. The results indicate that the CP is the most preferable grasping point from the viewpoint of stable grasping.

## 5. Conclusions

This study proposes a novel strategy for delicately grasping fragile objects using fluid fingertips. While pushing a target object at a constant speed, the fluid pressure behavior changes at the CP. This strategy advocates grasping at the CP because fractures of the target object can be avoided, while a substantial force or pressure can be transmitted to the target object by the fluid fingertips. Subsequently, we present a method for detecting the CP by comparing the fitting accuracies of fitting models. The features of this method are as follows: (1) online detection can be achieved, (2) only information on the fluid pressure is required, and (3) the threshold for the detection is constant with respect to the type of target object. The proposed method does not require advanced knowledge on the stiffness and deformation of the target object. The validity of the proposed approach is verified through several experiments.

The fluid pressure of the fingertips corresponds to the contact pressure, and thus, it includes object state information such as stiffness or viscosity. Therefore, the monitoring and regulation of the fluid pressure can provide the tuning of the fingertip stiffness according to the object state, leading to sophisticated grasping or manipulation strategies with the identification of unknown parameters, such as stiffness or viscosity, of an unknown target object. One example is the proposed strategy, in which the parameter or characteristic related with fracture was identified for delicate grasping. With the identification, the proposed strategy provided soft-contact impact with the target object along with enough fingertip stiffness for supporting the object weight via the tuning of the fingertip stiffness by the fluid pressure control. Our future work may involve the development of other types of grasping or manipulation strategies utilizing the fluid pressure control.

## Appendix A

We consider approximating a measured dataset $D=\left\{\left(x_{1},y_{1}\right),\left(x_{2},y_{2}\right),\ldots ,\left(x_{N},y_{N}\right)\right\}$ by the following d-dimensional polynomial function:

(A1) $$f^{\left(d\right)}\left(x\right)=\overset{d}{\underset{j=0}{\sum }}a_{j}x_{i}^{j}$$

The coefficients $a={\left[a_{0},a_{1},\ldots ,a_{d}\right]}^{T}$ are obtained by minimizing the following S:

(A2) $$a=\underset{a}{\mathrm{argmin}}S$$

(A3) $$S=\overset{N}{\underset{i=1}{\sum }}{\left(y_{i}-\overset{d}{\underset{j=0}{\sum }}a_{j}x_{i}^{j}\right)}^{2}$$

To minimize S, the following equation should be satisfied:

(A4) $$\frac{\partial S}{\partial a_{j}}=0$$

Subsequently, we have that

(A5) $$\left[\overset{N}{\underset{i=1}{\sum }}x^{j},\overset{N}{\underset{i=1}{\sum }}x^{j+1},\cdots ,\overset{N}{\underset{i=1}{\sum }}x^{j+d}\right]a=\overset{N}{\underset{i=1}{\sum }}y_{i}x_{i}^{j}$$

If we aggregate (12) for all j, we have the following:

(A6) $$\left[\begin{array}{cc} \begin{array}{cc} s_{0} & s_{1}\\ s_{1} & s_{2} \end{array} & \begin{array}{cc} \cdots & s_{d}\\ \cdots & s_{d+1} \end{array}\\ \begin{array}{cc} \vdots & \vdots \\ s_{d} & s_{d+1} \end{array} & \begin{array}{cc} \ddots & \vdots \\ \cdots & s_{2d} \end{array} \end{array}\right]a=\left[\begin{array}{c} t_{0}\\ t_{1}\\ \vdots \\ t_{d} \end{array}\right]$$

Here,

(A7) $$s_{m}=\overset{N}{\underset{i=1}{\sum }}x_{i}^{m},\;t_{m}=\overset{N}{\underset{i=1}{\sum }}y_{i}x_{i}^{m}$$

$s_{m}$ and $t_{m}$ are defined by the dataset. Therefore, ***a*** is obtained by the following equation:

(A8) $$a={\left[\begin{array}{cc} \begin{array}{cc} s_{0} & s_{1}\\ s_{1} & s_{2} \end{array} & \begin{array}{cc} \cdots & s_{d}\\ \cdots & s_{d+1} \end{array}\\ \begin{array}{cc} \vdots & \vdots \\ s_{d} & s_{d+1} \end{array} & \begin{array}{cc} \ddots & \vdots \\ \cdots & s_{2d} \end{array} \end{array}\right]}^{-1}\left[\begin{array}{c} t_{0}\\ t_{1}\\ \vdots \\ t_{d} \end{array}\right]$$
